# Operation Under High Ionizing Dose Rates of Gamma or X-Ray Radiation of a 10 µm Radiation Tolerant Global Shutter Pixel †

**DOI:** 10.3390/s25226979

**Published:** 2025-11-14

**Authors:** Pedro Santos, Idham Hafizh, Paul Leroux, Guy Meynants

**Affiliations:** ADVISE Lab, KU Leuven, Department of Electrical Engineering (ESAT), Geel Campus, Kleinhoefstraat 4, 2440 Geel, Belgium

**Keywords:** CMOS image sensors, total ionizing dose (TID), global shutter, X-ray, gamma radiation

## Abstract

A 10 × 10 µm^2^ radiation-tolerant voltage-domain global shutter pixel with radiation-hardened by design (RHBD) device modification is developed to operate under high ionizing-dose rates and high total ionizing-dose (TID) levels. Therefore, a modified NMOS transistor layout is used in the pixel to achieve radiation hardness. The pixel design is demonstrated to operate up to 1 MGy or 100 Mrad (SiO_2_) TID with minimal degradation. The global shutter pixel also includes correlated double sampling (CDS) to reduce noise and the impact of the collected carriers generated by the flux of gamma or X-ray radiation. Combined with an external flash, global shutter operation allows short exposures, which limits the impact of radiation on dark current and dynamic range. The pixel is designed using 180 nm CMOS Image Sensor (CIS) technology.

## 1. Introduction

Nowadays, CMOS Image Sensors (CISs) are required in various applications: from consumer, industrial, medical, to scientific [1]. For special applications, such as ionizing radiation environments, CIS are the most promising type of photodetectors for performance imaging applications where radiation hardening is required: space, nuclear, scientific, medical, or even military operations. Multiple solutions have been proposed in recent years with rolling shutter 3T or 4T pixel architectures [2,3,4,5,6]. However, rolling shutter operation presents some limitations, like acquisition speed, motion artifacts, or synchronization.

Global shutter pixels also support flash illumination with a short exposure time [7], which compensates for the contribution of directly generated carriers from gamma or X-ray radiation through Compton scattering or the photoelectric effect [3,5,6,8] to the photosignal. The result is a lower noise operation with short exposure and limited degradation with TID.

### Radiation Effects on Electronics

Electronic devices and, therefore, CMOS transistors are impacted by ionizing radiation through the cumulative creation of positive charges in the SiO_2_ dielectric layers and an increase in interface states at the Si–SiO_2_ interface. These lead to a shift in the transistor threshold voltage (Vth), a degradation of the carrier’s mobility (*µ*), the increase of 1/*f* noise, and the formation of a parasitic source–drain leakage path via the STI and field in the *n*-MOSFET.

In CMOS pixels, these effects lead to an increase in output offset and 1/*f* noise, lateral shunting between pixels, lower gain, a photon response non-uniformity (PRNU) increase, and an average dark current (*I_dark_*) increase, and therefore, dark signal non-uniformity (DSNU). The *I_dark_* increase, especially at low-TID levels, is dependent on the presence of sidewall trench isolation (STI) along the channel, and due to the increase in interface state density, combined with the depletion region modification close to the surface [9]. To limit the impact of TID effects, new device layouts were studied. Vth shift, 1/*f* noise, and *µ* cannot be improved just by design since they rely on technological parameters: the thickness of SiO_2_, the W/L of the transistor, and the doping concentrations. Therefore, the focus is on minimizing the source-to-drain leakage, which significantly impacts the pixel performance. Considering the enclosed layout transistor (ELT) [9,10], there is no STI along the channel, and the connection between the drain and the source cannot be built, making this a good option to be considered. ELT devices, however, require more area compared to straight devices. Being fully enclosed, the gate is significantly larger, the channel under it is also greater, and therefore, the W/L ratio is larger than the smallest straight devices in the same technology. Additionally, the outer terminal—typically the drain in *n*-MOSFET—is unshielded and requires a guard ring to avoid leakage to neighbor transistors. All of these constraints require space, which is critical in pixel design, especially for small pixel pitch designs.

**Figure 1:**
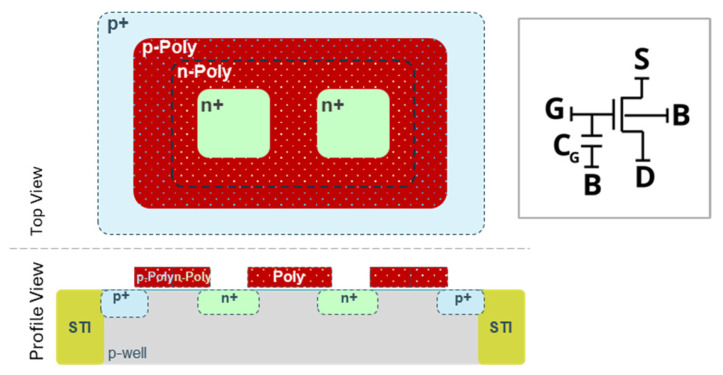
Butterfly (BF) NMOS Device, a compact enclosed device. A simple device schematic is presented with the additional C_G_ reflected, representing the additional gate capacitance of the enclosed device.

An alternative to ELT devices, and yet an enclosed device benefiting from all its advantages, is the butterfly (BF) MOSFET (Figure 1) [9]. This is a fully enclosed device with a compact layout scheme and a small W/L ratio. Since both drain and source are fully enclosed with a polysilicon gate, no lateral STI is present, and thus, there is no formation of leakage paths up to high-TID levels. To increase the radiation tolerance for very high TID, a *p*+ implant is deposited from the outer half side of the polysilicon gate enclosure.

For 180 nm CMOS technology and for higher-than-1 MGy TID (SiO_2_), CMOS devices have been measured with Vth shifts of +0.15 V in NMOS and −1.0 V in PMOS, respectively. In addition to this deviation, and for the same technology, up to 40% mobility degradation is expected in devices with a channel length below 0.44 µm [9,10]. Due to these reasons, no minimal-size BF transistor was used in this work.

## 2. Global Shutter Pixel with RHBD Devices

The pixel is designed with “butterfly” (BF) NMOS transistors, eliminating parasitic field leakage currents by design. As discussed, for pixel arrays, the most critical aspect is area, and the pixel electronics are expected to be as small as possible to guarantee the maximum fill factor. The BF transistor is a fully enclosed device with a compact layout scheme and a small W/L ratio.

**Figure 2:**
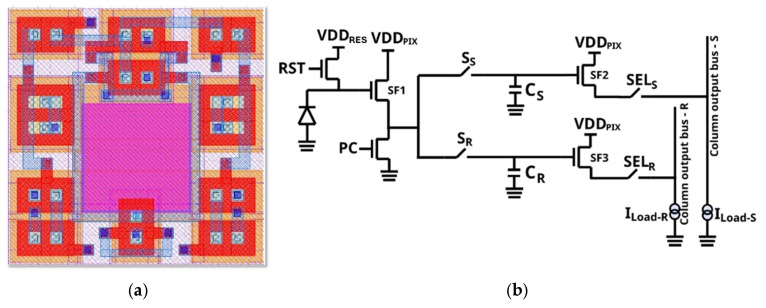
(**a**) Global shutter layout proposal. (**b**) Schematic of the global shutter pixel.

**Figure 3:**
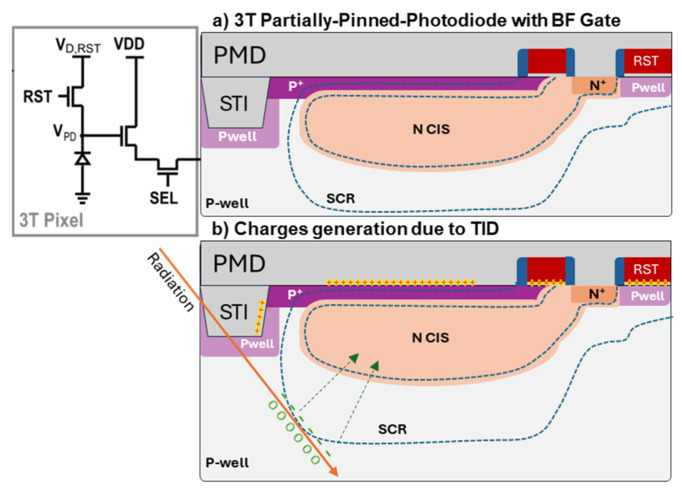
A 3T Pixel profile for radiation conditions: (**a**) PD profile in pre-rad condition; (**b**) PD profile in rad conditions with charge buildup and the Si–SiO_2_ interfaces due to TID and modification of depletion region.

The pixel architecture (Figure 2a,b) has nine transistors and two capacitive storage nodes. All the devices are BF-type devices, including the storage nodes. The pixel is tested up to 1 MGy (100 Mrad) TID (SiO_2_) with X-ray radiation. To withstand such a high TID value, a partially pinned photodiode is used (Figure 3a [11]). The intentional use of a strong *p*+ implant on the photodiode and recessed shallow trench isolation (STI) reduces the generation centers from the TID-induced interface traps at SiO_2_ interface. In-pixel storage nodes or memories are implemented using enclosed MOS capacitors, which are also inherently radiation-tolerant by design.

Although efforts were made to reduce leakage paths and shield the pixel as best as possible, for very high TID levels, there are still leakage paths, which represent the additional dark current. These paths, as introduced, have three main sources [12,13,14]: the depletion dark current generated in the PD depletion region, the diffusion dark current generated in the field-free region, and the surface dark current generated on all the Si–SiO2 interfaces (Figure 3b). The in-pixel storage nodes also generate dark current due to switch junction leakage. Since the storage capacitances are relatively large, the use of CDS to mitigate the signal injection and the impact of TID in those devices is limited; the focus of our study is on the PD sources. In the proposed design, the significant recession of the STI and the RHBD transistor used are lower contributors compared to the PMD interface above the PD. Additionally, the trapped charges and interface states present in the source-follower gate transistor also contribute to dark signal non-uniformity (DSNU).

Another aspect that is important to note is the presence or absence of radiation flux and its contribution to dark signal and DSNU. Without a flux of ionizing radiation, the mentioned leakage paths are the main contributors to the dark signal and DSNU. Thus, with very high TID levels, the DSNU also increases due to the random nature of positive charge generation and interface traps, proportional to the radiation flux that crosses the PD region over time. With radiation flux present, besides the dark current source buildup due to very high TID, the PD depletion region will integrate the charges generated from the Compton scattering or the photoelectric effect due to the presence of the high-energy particles or photons from the radiation source that crosses the PD and the field below. This electron generation will add up to the dark current paths, dramatically increasing the dark signal amplitude. The DSNU is largely increased as well due to the stochastic nature of the radiation flux.

The photodiode (PD) connects to the gate of the source follower (SF1) and the drain of the reset switch (Figure 2b). To read the SF1 voltage, an active load or precharge (PC) is used. The pixel reset voltage—when PD is being reset—and pixel signal voltage—at the end of the PD exposure—are stored in two storage nodes: the C_R_ and C_S_ MOS capacitors, respectively. For this storage, dedicated switches (S_R_ and S_S_) are used to connect to two independent output buses. The proposed pixel architecture, unlike state-of-the-art non-radhard pixels, does not use a transfer gate (TG), and does not feature in-pixel charge transfer. For very high TID (>>10 kGy TID), the charge transfer degrades with TID due to a significant charge transfer efficiency (CTE) drop and lag increase, caused by a drop in carrier mobility, *µ*, and due to charges built up in oxide and spacer regions [11,15,16]. For the same reasons, a charge domain global shutter architecture was also not considered for this work.

The pixel is designed using 180 nm CMOS Image Sensor (CIS) technology. Currently, X-ray radiation is used for TID accumulation. Future Co^60^ experiments are considered for radiation degradation investigation purposes.

## 3. Experimental Results

**Figure 4:**
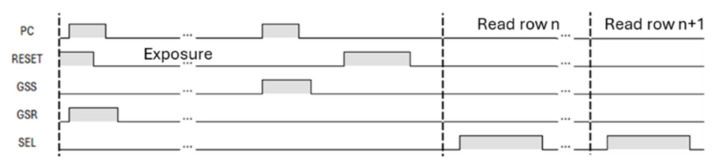
Timing scheme of the global shutter pixel.

Testchips with the presented GS pixel architecture were evaluated before and after irradiation. The results and the respective discussion are presented in this section. The testchip includes a small matrix of 8 × 8 pixels. The applied timing is presented in Figure 4, and the supply levels are VDDRES = 2.8 V and VDDPIX = 3.3 V. The testchips have independent analog outputs for pixel signal and pixel reset values (Figure 4).

The acquisition system uses a dual 14-bit resolution ADC that acquires the pixel signal and pixel reset simultaneously. The conversion step of the ADC is 152.7 µV/DN. For all testchips and all measurement conditions, the PD is operated in hard reset to avoid low illumination non-uniformity.

There were also two process-split lots available for comparison. The original split includes all the standard CIS process doping, while in the second split lot, the photodiode implant is less abrupt, which can reduce the dark current after radiation. In this article, the original split is named OR, and a second split lot is named the SP. The individual device name conventions are ORxx and SPxx, where xx is the device number.

Testchips were measured before and after radiation steps in multiple setups and temperature conditions. The X-ray equipment is an XRD diffraction tube with a tungsten target material that produces high-energy photons with a peak spectrum at 10 keV. The produced dose rate for this experiment was 3.6 kGy/h (100 rad/s) for the entire radiation campaign.

Two testchips were irradiated up to 1 MGy TID (SiO_2_), where during HTA, the sample SP02 was kept unbiased, while the sample SP03 was kept biased. For the SP split, the highest TID level is 1 MGy TID (SiO_2_), followed by a high-temperature annealing (HTA). The HTA consists of keeping the device biased or unbiased at a constant temperature of 100 °C for 7 days (168 h). The OR split was irradiated just until 580 kGy and followed by an unbiased annealing step as described.

**Figure 5:**
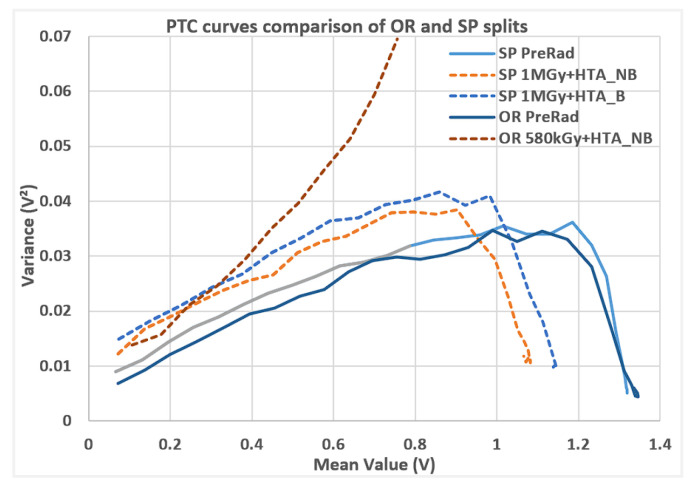
PTC curves comparison of OR and SP splits, before and after high TID levels (SiO_2_). Pre-rad curves show similar results; however, for the radiated devices, OR split shows greater degradation than SP splits due to increased dark current.

The photon transfer (PTC) curves (Figure 5) show the signal–noise relation with the split lot and the TID levels for the different devices. The biggest positive impact of radiation hardness is observed on the split lot. Regardless of the BF transistor implementation in the pixel, the photodiode implant doping with a higher concentration (OR split) generates a significant dark current after radiation.

This is due to higher doping, resulting in an abrupt *n*+/*p*+ junction and, therefore, higher dark signal with TID, enhanced by the strong electric field over the junction. The OR split already shows too-large degradation at half of the target TID level (580 kGy). The SP split, on the other hand, has a marginal impact after 1 MGy TID (SiO_2_). Additionally, the peak point of PTC (which is a metric for full-well charge of the pixel) after radiation and for SP split shows a reduction in just around 20%. This is not due to FWC reduction on the photodiode, which remains constant, but due to an output swing reduction caused by the readout transistor’s Vth shift.

**Figure 6:**
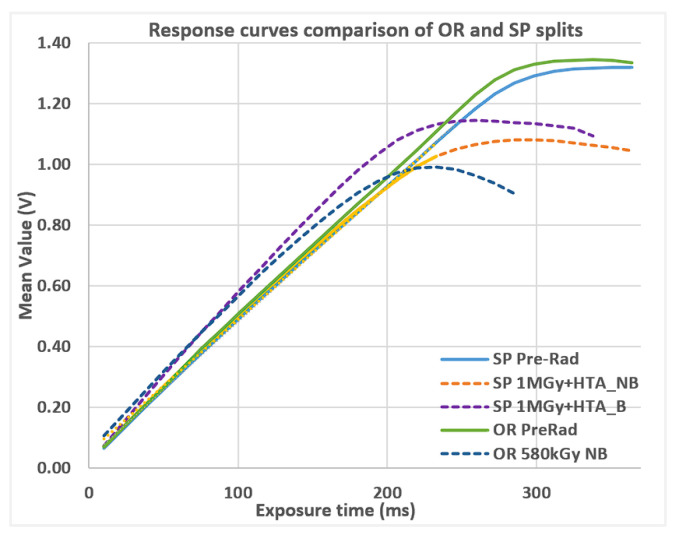
Response curves comparison of OR and SP splits, before and after high TID levels (SiO_2_). After radiation, the response is reduced, mainly due to the readout reduced output swing.

The response curves (Figure 6) are in line with PTC. SP lot presents almost no degradation, except in the output swing reduction. This is due to Vth shifts as introduced in Section 1, limiting the working range of the pixel itself. This is true for pixel source followers and for the testchip readout circuit, where both NMOS and PMOS transistor-based circuits are present and where TID effects limit the operational voltage swing.

### Dark Current and Radiation

Another important aspect to consider is the dark current (*I_dark_*) evolution with very high TID and its dependence on temperature (*T*). As proposed in [10], *I_dark_* is expressed in *e*^−^/*s* and its evolution with temperature can be expressed as follows:

(1) $$I_{dark}=I_{0}\cdot e^{-\frac{∆E}{k\cdot T}}$$

where *I*_0_ is the dark current, Δ*E* is the bandgap activation energy, *k* is the Boltzmann constant, and *T* is the temperature. The equation can also be rearranged as follows [12,17]:

(2) $$I_{dark}=I_{0}\cdot e^{a\cdot T}$$

where *a* can be treated as a constant for a given temperature, which includes the bandgap activation energy. For a silicon substrate, the typical activation energies for dark current generation are typically Δ*E* = 1.1 *e* − *V* for diffusion and Δ*E*/2 = 0.6 *e* − *V* for depletion.

**Figure 7:**
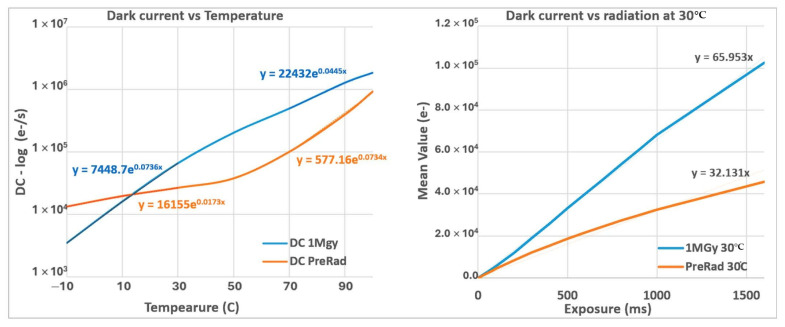
Dark current figures of the SP split before and after radiation with temperature (**left**) and with exposure time (**right**) with T = 30 °C. The radiation effects changed the behavior of dark current with temperature due to radiation effects on SiO_2_ interfaces.

For the current work, the dark current evolution with temperature and radiation is presented in Figure 7. On the left side, the evolution of dark current with temperature is shown before and after X-ray irradiation. On the right side, the dark current evolution with the exposure time of the SP split devices is shown at a constant temperature of 30 °C, and for the radiated and non-radiated devices.

Concerning the pre-rad condition and in accordance with Equation (1), the dark current curve follows an exponential relationship with temperature. Thus, two sections of the graph are easily identified: below ~50 °C, where the depletion dark current is the dominant contributor, and above ~50 °C, where the diffusion dark current is the dominant contributor.

With radiation and the TID, the response and behavior changes: the main contributors are no longer just the diffusion or just the depletion, but the surface dark current due to charges and interface state buildup at the Si–SiO_2_ interface above the PD. Although due to the nature of these currents, the *I*_0_ component of Equation (1) is the dominant contributor. In other words, with the increase in TID to extreme levels, the depletion region changes, leading to an increase in dark current. Combined with interface state density, the variation in the dark current with temperature is significantly modified: the increase in TID levels results in an electric field modification, leading to an increase in collected charges.

Summarizing, according to Equation (2), the pre-rad value of a is, as follows:

*a_depletion_* = 0.017 °C^−1^ and *a_diffusion_* = 0.07 °C^−1^

which is consistent with the activation energy variation with temperature of the silicon bandgap [12,17]. For the post-radiated devices, the values of a are, as follows:

*a*_<50 °C_ = 0.0736 °C^−1^ and *a*_>50 °C_ = 0.0445 °C^−1^

which suggests that the contribution of electron migration due to activation energy is less dominant with very high TID and depletion-region modification, resulting in a dark current behavior similar to dark current diffusion, but with a greater signal contribution from the Si–SiO_2_ interface as previously introduced.

Concerning the (Figure 7; right), with a fixed temperature of 30 °C, it is possible to observe that the TID doubles the *I_dark_* compared to the pre-radiation condition. This observation also confirms that after radiation, the carrier contribution is modified, with the dominant contributors being the dielectric interface states and Si–SiO_2_ interface.

**Figure 8:**
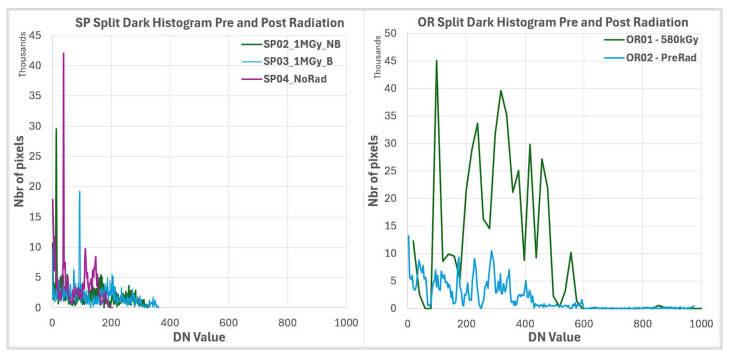
Dark signal distribution at room temperature for OR and SP splits, before and after radiation campaigns. SP split shows a better dark signal histogram for before and after TID, due to an improved PD implant.

Dark signal distribution histograms are considered in Figure 8. This experiment was performed at room temperature. Due to the limited number of pixels, a set of 50 frames was taken to build the histograms. It is noticeable that there is an evolution of the dark signal from before to after the radiation campaign in both process splits. Additionally, the SP split presents a better dark signal distribution with a smaller tail part, even before radiation. Additionally, the SP split presents limited dark signal degradation due to radiation effects.

**Figure 9:**
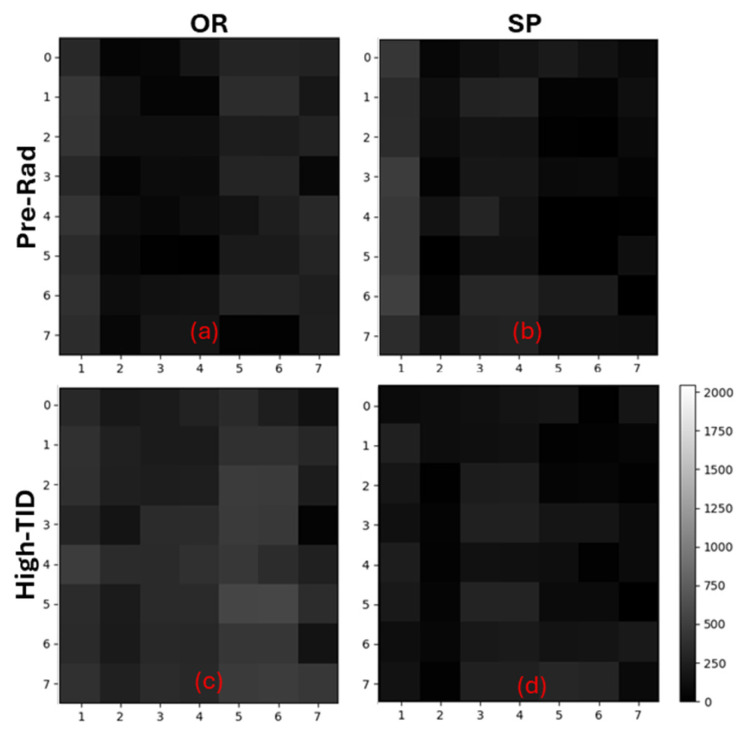
OR and SP split dark images with 10 ms exposure and 8× gain. (**a**) OR split pre-rad; (**b**) SP split pre-rad; (**c**) OR split 580 kGy TID (SiO_2_); (**d**) SP split 1 MGy TID (SiO_2_).

Figure 9 presents images in dark conditions from OR (left side) and SP (right side) splits before and after the radiation campaign, with a gain of 8× and 10 ms exposure time. The right-side scale presents values in DN. The non-radiated condition shows similar results and noticeable column FPN. The presence of a relatively strong column FPN is due to the readout columns being a radiation-hardened design without a common reference signal or offset compensation, resulting in a considerably strong column FPN, mainly due to device mismatch. This FPN is expected to increase with TID due to the increase in transistor mismatch. The images from the irradiated devices (c) and (d) have some visible degradation; in particular, the OR split (left) after 580 kGy TID (SiO_2_), which shows a more noticeable degradation of the pixel response.

It is also possible to observe a strong DSNU, besides the column FPN. With TID increase, multiple mechanisms take place: first—the radiation-induced defects have a non-homogeneous distribution across the pixel photodiode regions, enhancing the dark current non-uniformity (DCNU); second—the pixel transistors mismatch increases, leading to a greater signal non-uniformity when compared with before-radiation.

## 4. Discussion

Table 1 presents a numerical compilation of the measured parameters of the measured devices. The conversion step of the ADC is 152.7 µV/DN. The first important fact is that for this particular demonstrator, the CDS cannot completely eliminate the read noise, and therefore, it is considerably higher before radiation.

There are two reasons to explain this. First, the pixel is based on 3T architecture, and thus, the PD cap results in greater *kTC* and 1/*f* noise, resulting in a large read noise. This capacitance is estimated to be C_PD_ ≈ 16.6 fF, including parasitics, leading to a *kTC* noise of 74 e-_RMS_; second, the storage capacitance C_SS_ = C_SR_ is estimated to be 21 fF each, or just slightly larger than the C_PD_, meaning that the non-negligible kTC noise of the storage capacitance is also included in the generated signal. Thus, the resulting calculated read noise is around 146 e-_RMS_. Pixel temporal noise would be considerably reduced with larger storage capacitors, as can be offered by deep trench capacitors in modern CIS processes. However, comparing the read noise before and after TID, especially of the SP split, the increase is just marginal.

Given the TID values of the OR split, the degradation observed is too great by the test results, and therefore, will not be considered as a possible solution.

For the SP split lot, the conversion gain variation is also minimal from before to after radiation. This means that marginal gain variation is expected with the present architecture for this split. Additionally, for the SP split, the FWC is kept close to 175 ke^−^, as expected.

The dynamic range reduction is due to the output swing reduction, mainly due to the Vth shift and mismatch increase in all transistors in the pixel and readout chain.

Another important parameter regarding the global shutter pixels is the Parasitic Light Sensitivity of the storage nodes (PLS), defined as the ratio of sensitivity of the storage nodes over the sensitivity of the photodiode, after CDS. Both splits have a reasonable PLS before radiation (1/22,000 or better), which drops to 1/7400 after 1 MGy and unbiased thermal annealing. The leakage current on the storage capacitors increases from 0.8 mV/s pre-rad to 9.98 mV/s after 1 MGy (SiO_2_). Most of this increase is canceled after (correlated) double sampling since it is similar on both capacitors.

## 5. Conclusions

The 10 × 10 µm^2^ voltage-domain global shutter pixel uses device-level modifications that effectively mitigate total ionizing dose (TID) effects and read noise increase by using correlated double sampling (CDS).

The use of the BF device modification enabled the required radiation tolerance for the TID levels up to 1 MGy (SiO_2_). The photodiode modifications: partially pinned, recessed STI, and PD implant reduction, show an increase in robustness to TID and moderate I_dark_ increase.

The presented GS pixel SP split, utilizing a less abrupt photodiode junction, shows radiation tolerance to up to 1 MGy TID (SiO_2_) with marginal degradation.

Dark current evolution and dependency on temperature show a deviation from the exponential curve of the pre-radiation devices. This is due to the dominant contribution of the surface leakage paths over the PD region, being the main dark current contributors for very high TID levels.

The pre-radiation results are modest compared to the state of the art, as the main focus of this development is on high-TID effects tolerance. Therefore, the study focused on implementing a novel RHBD strategies that achieve the primary end goal, being conservative concerning pre-radiation noise performance.

Future designs should improve read noise and seek improved photodiode architectures and true CDS operation, resulting in radiation-hardness and low-noise pixel architecture.

## Figures and Tables

**Table 1:** Summary of pixel parameters with TID and splits.

Parameter	OR Pre-Rad	OR 580 kGy	SP Pre-Rad	SP 1 MGy + HTA N Bias	SP 1 MGy + HTA Bias	Units
Read noise	164.4	360	139.8	200.2	255.4	e-_RMS_
Conversion gain	5.30	10.49	5.96	5.64	5.89	µV/e^−^
FWC	198,001	90,023	172,049	176,245	175,980	e^−^
Dynamic Range	61.61	57.3	61.80	58.7	57.69	dB
DSNU	361	429	297	303	311	e^−^
PLS	0.0046	0.052	0.0023	0.014	0.009	%
	(1:22,000)	(1:1950)	(1:43,000)	(1:7400)	(1:11,300)	

## Data Availability

Data are contained within the article.
